# Insulo‐opercular stereoelectroencephalography exploration in children and young adults: Indications, techniques, and safety

**DOI:** 10.1002/epi4.12651

**Published:** 2022-10-05

**Authors:** Akanksha S. Chilukuri, Emefa Awkwayena, Taylor J. Abel

**Affiliations:** ^1^ Department of Neurological Surgery University of Pittsburgh Pittsburgh Pennsylvania USA; ^2^ Department of Bioengineering University of Pittsburgh Pittsburgh Pennsylvania USA

**Keywords:** epilepsy, operculum, pediatric, sEEG

## Abstract

**Objective:**

Sampling the insulo‐opercular region with invasive recordings is crucial given the importance of this region in epileptic networks and a variety of electroclinical presentations. However, implantation of the insulo‐opercular region via stereoelectroencephalography (sEEG) is considered technically challenging given complex vascular and gray matter relationships in this region. We investigated the safety of insulo‐opercular sEEG exploration in children and young adults using standard sEEG approaches: (1) orthogonal insulo‐opercular (including the pseudo‐orthogonal insulo‐opercular approach) and (2) medial‐lateral insular oblique approach.

**Methods:**

We performed a retrospective cohort study of 30 consecutive patients who underwent 33 sEEG implantations. All patients had drug‐resistant focal epilepsy, were between the ages of 4 and 21, and were operated at one institution between January 2019 and March 2021. Medical records and neuroimaging were reviewed. Hemorrhage, infection, and other complications were considered as outcome variables.

**Results:**

A total of 519 electrodes were placed. Eighty‐one were placed orthogonally into the temporal operculum, 53 orthogonally into the frontal operculum, and 19 obliquely into the insula. sEEG electrodes localized seizure onset to the insulo‐opercular region in eight patients, leading to a resection three times, an ablation four times, and one peri‐insular hemispherectomy. Of the 519 electrodes placed, none of them exhibited hemorrhage or serious complications. Of the 153 electrodes placed into the insula, none had any permanent deficits or complications and one had minor bleeding due to the electrode breaking.

**Significance:**

These results demonstrate that the orthogonal (including pseudo‐orthogonal) and medial approaches to sampling the insula are safe.

**Key Points**

- Implanting orthogonal and medial‐lateral oblique sEEG electrodes resulted in no permanent complications in pediatric epilepsy patients.
- sEEG is a useful monitoring tool that often affects surgical decision making.
- Future work is necessary to determine seizure freedom in various invasive monitoring methods.

## 1 INTRODUCTION

Identification of the epileptogenic zone (EZ) is key to planning safe and effective resective epilepsy surgery. In 30%–50% of patients, however, non‐invasive measures fail to delineate the EZ.^1^ Stereoelectroencephalography (sEEG) is a method that provides ictal and interictal recordings via the implantation of intracerebral electrodes to validate and further define the EZ in epilepsy surgery candidates.^1^, ^2^

Sampling the insulo‐opercular (IO) region with invasive recordings is crucial given the connectivity of this region to multiple brain regions in a variety of electroclinical presentations,^3^, ^4^, ^5^ including insulo‐opercular epilepsy, temporal‐plus epilepsy involving the IO region, and ipsilateral insula‐insula connections that increase integration.^6^, ^7^, ^8^ Temporal‐plus epilepsy, defined as having EZ spread over the temporal lobe and surrounding brain regions, results in lower rates of seizure freedom than typical temporal lobe epilepsy.^9^ In such cases and when the EZ is in deeper brain structures, sEEG has been shown to be a useful tool in identifying the EZ.^10^

However, the ideal approach for implantation of the IO region is still a matter of debate.^11^, ^12^, ^13^ Only in the past few decades have sEEG and surgical interventions in the insulo‐opercular region resulted in favorable seizure outcomes.^14^, ^15^, ^16^

Instrumentation of the IO region with electrodes can be technically challenging given the folds of the sylvian fissure, the complex orientation of cortical gray matter, and the venous and arterial structures passing through this region.^17^ Several technical approaches to implanting electrodes in this region have been advanced including utilizing a posterior entry point at the parieto‐occipital junction with contact placement parallel to the insular cortex^18^ and an open method of splitting the sylvian fissure or using a transsylvian approach,^19^ but there is an ongoing debate about the safety and efficacy of different recording approaches. The orthogonal and medial‐lateral insular oblique approaches enable exploration of the insulo‐opercular gray matter and seem to be safe in retrospective observational studies.^20^, ^21^, ^22^ However, less is known about the application of these trajectories in pediatric epilepsy surgery patients.^3^, ^11^

This study investigates the safety of insulo‐opercular sEEG exploration in children and young adults using the (1) orthogonal (or pseudo‐orthogonal) insulo‐opercular approach and (2) medial‐lateral insular approach.

## 2 METHODS

We performed a retrospective review of medical records using an IRB‐approved protocol. Given the retrospective and minimal risk nature of the study, consent requirements were waived. All implantations were performed by a single surgeon. Our sEEG implantation technique is similar to that previously described.^23^

### 2.1 Patient selection

We included 30 patients with 33 SEEG implantations from drug‐resistant focal epilepsy patients ages of 4 and 21 that were operated at a single institution between January 2019 and March 2021.

### 2.2 Implantation methods

To prepare for surgery, any patients on anticoagulants or antiplatelet agents are discontinued and epilepsy medications are replaced with appropriate medication. Medications are tapered appropriately in the weeks leading up to surgery to induce and record spontaneous seizures. All patients then undergo a preimplantation neuroimaging protocol that includes (1) high‐resolution postcontrast MRI (magnetization‐prepared rapid acquisition gradient echo [MPRAGE] sequence) and (2) thin‐slice postcontrast Robotic Stereotactic Assistance (ROSA)–protocol CT.

The sEEG implantation strategy is designed with the anatomo‐electro‐clinical hypothesis, which is formulated based on clinical semiology, neuropsychology, MRI findings, and long‐term video EEG recordings in mind.

Patients are brought to the OR and undergo general anesthesia. A stereotactic frame with 4 head fixation points (Leksell frame) is used to position the head and ensure accuracy of stereotactic implantation. Then, bone fiducials are inserted using a 3D rendering from the ROSA software to avoid overlap with electrode trajectories and used as fiducial markers. Next, an intraoperative CT (O‐Arm; Medtronic) is obtained and coregistered to the preoperative stereotactic CT by using the ROSA software. An error measurement <0.8 mm is considered acceptable. The bone fiducials are maintained in position after electrode implantation in case the patient is taken back to the OR for further electrode implantation.

**FIGURE 1:**
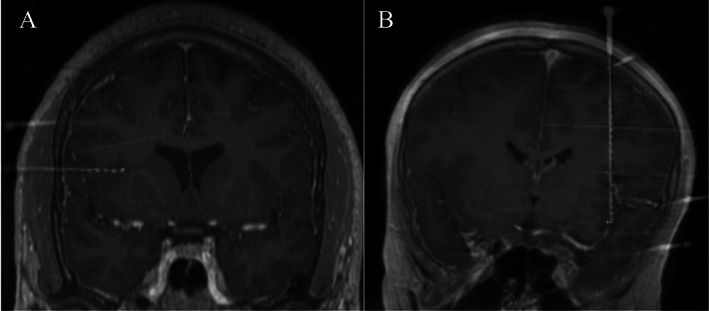
A, Example of the orthogonal approach to the IO. B, example of the oblique approach to the IO

The robotic implantation is initialized, and the first trajectory is selected. (Figure 1) Through the robotic guidance instrument, a power drill is used to enter the skull directly through the scalp. Bone debris is irrigated from the skull opening and a sharp coagulation electrode is inserted to open the dura mater. A guidance screw (20, 25, 30, or 35 mm, with the length depending on the depth of bone) is inserted into the skull. A stylet measured to the length of the target is then inserted to initiate the electrode trajectory. Care is taken to minimize the egress of CSF after opening the dura, but prior to electrode implantation, because this may result in slight alterations of brain position. Finally, the electrode is inserted directly through the guidance screw and is then secured to the screw at its final position. For all patients we used either AdTek (1.1 mm diameter), PMT (0.86 mm diameter), or Dixi (0.8 mm diameter) sEEG electrodes.

An intraoperative O‐Arm CT is obtained to confirm electrode positions. This is then co‐registered to the preoperative CT using the ROSA software. The electrode positions are adjusted as necessary after comparison to preplanned positions on the preoperative MRI and adequate coverage of gray matter is assured.

SEEG recordings begin a few hours after implantation. The children are monitored carefully, and antiepileptic drugs are carefully tapered to induce seizures. The electrodes are then removed under general anesthesia after the EZ is successfully localized.

## 3 RESULTS

### 3.1 Patient characteristics

A summary of patient characteristics and total electrodes placed is given in Table 1. All patients had drug‐resistant epilepsy and most had a seizure frequency of daily or weekly. There are a total of 30 patients and 33 sEEG implantations. Eight patients had repeat surgeries, where three were repeat sEEG, three patients had previous subdural grids placed and two of those had previous resections. The other two patients had ventriculoperitoneal shunts. Sixteen of the 33 interventions had a pathology diagnosis, in which the most common were focal cortical dysplasia and gliosis. No patients had lesions in the insula.

**TABLE 1:** Summary of patient history and interventions

# of interventions	33
Age of Onset	0 to 14 Mean: 6.23 SD: 4.62
Age at surgery	4 to 21 Mean: 13.42 SD: 4.53
% Female	52%
Previous surgeries %	24%
# of total electrodes	519
# of Temporal Orthogonal Electrodes	81 (23 patients)
# of Frontal Orthogonal Electrodes	53 (21 patients)
# of Insular Oblique Electrodes	19 (14 patients)
Laterality	R: 13 L: 7 B: 13
Pathology	DNET: 1 Oligodendroglioma: 1 (WHO 2) and 1 (WHO not assigned) Gliosis: 4 Tuberous sclerosis: 3 Juvenile pilocytic astrocytoma: 1 FCD: 4 Leptomeningeal fibrosis: 1 Rasmussen’s encephalitis: 2

Since January 2019, a total of 519 electrodes have been placed. Eighty‐one were placed orthogonally into the temporal operculum, 53 were placed orthogonally into the frontal operculum, and 19 were placed obliquely into the insula. Among the 30 pediatric patients, the age of onset had a range from 0 to 14 years with a mean of 6.23 years and standard deviation of 4.62. The range of ages at implantation are 4‐21 with a mean of 13.42 and standard deviation of 4.53.

### 3.2 Localization of the EZ

**FIGURE 2:**
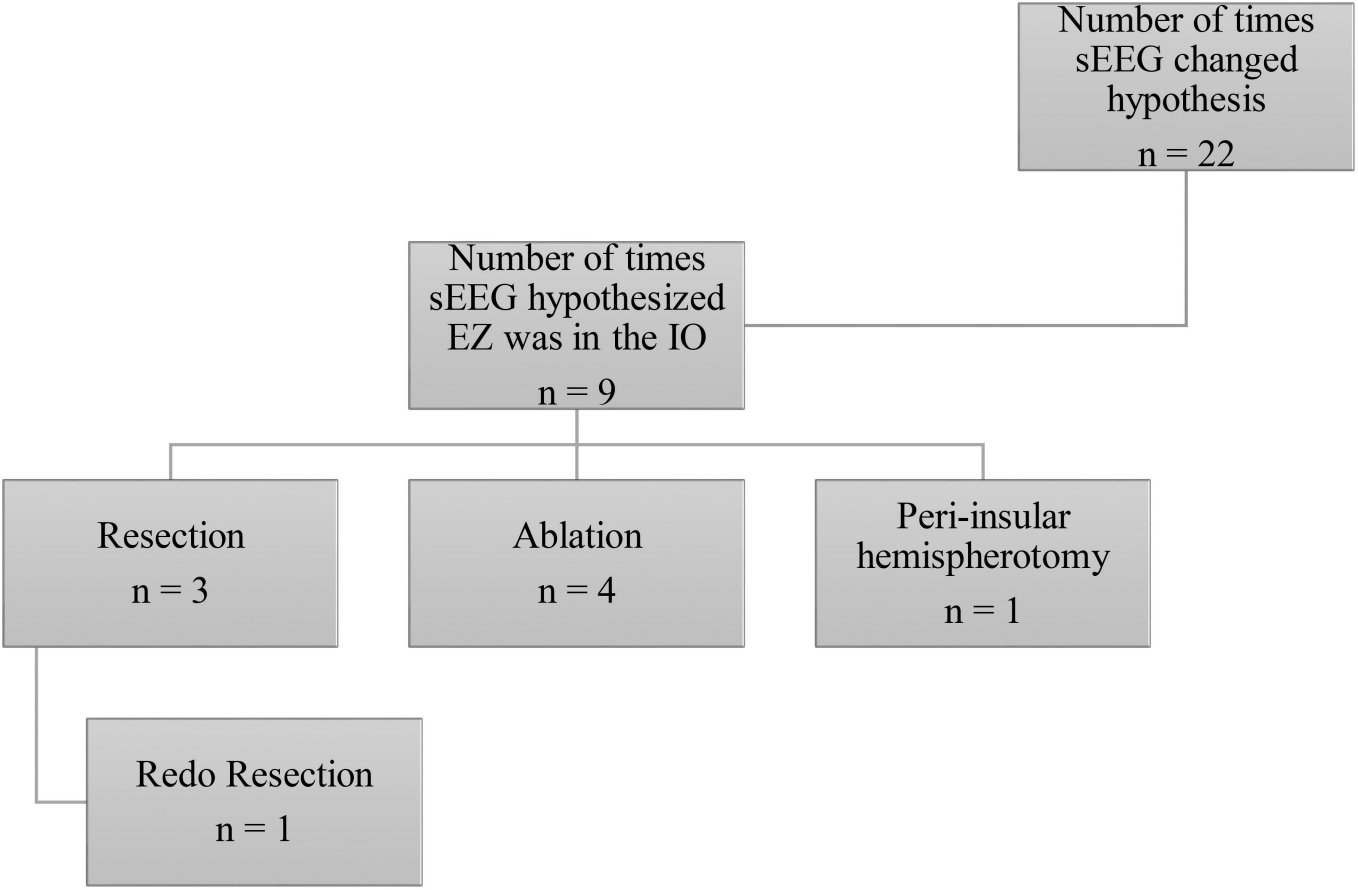
How impactful sEEG was in changing the hypothesized EZ, specifically in the IO region and follow‐up interventions

**FIGURE 3:**
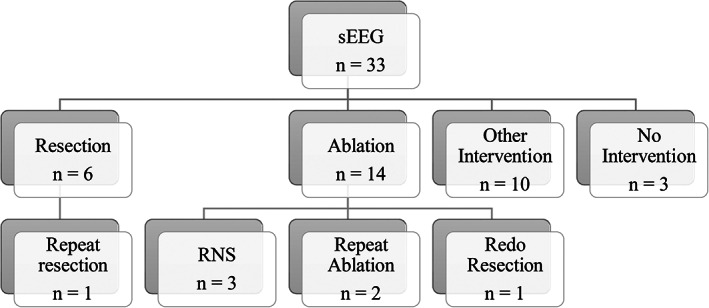
Interventions chosen after sEEG monitoring for all patients

Figure 2 shows where the hypothesized EZ is before and after sEEG placement surgery. Results show that sEEG placement helped narrow the EZ almost every time and was able to alter the surgical plan 22 of 33 times. Of all the 33 interventions, 14 received an ablation and six received a resection. Of the remaining 13, one had a right cortico‐amygdalo‐hippocampectomy, two had peri‐insular hemispherotomies, four had an RNS placed, one had a VNS (and subsequent RNS), two had sEEG repeated and three had no intervention but two of those are RNS candidates. Of the 14 patients that had an ablation, three had an RNS placed later, two had a repeat ablation, and one had a redo resection so almost half needed an additional intervention. However, in the six patients that had a resection, only one needed an additional intervention of a redo resection 2 days later (Figure 3).

More interestingly for us, sEEG changed the hypothesis from or to the IO region 9 of the 22 total times the hypothesis changed and was able to localize seizure onset to the insulo‐opercular region eight times (Figure 2). Of those eight, a resection was performed three times, an ablation four times, and one had a peri‐insular hemispherectomy that was redone 5 months later. None of the patients that had an ablation needed an additional intervention done and only one patient that had a resection needed a redo resection 2 days later. Overall, of the eight patients that had an EZ localized to the insulo‐opercular region only two needed an additional intervention. Overall, of the 25 patients that had an epileptogenic zone localized outside of the insulo‐opercular region, 10 needed an additional intervention or were candidates to receive one soon (Figure 3).

### 3.3 Seizure outcomes

Overall, 20 patients have follow‐up seizure outcome data for at least 1 year and eight are still seizure free. Of the remaining 10 patients, three were seizure free at the latest time point which was anywhere from 0.17 to 0.92 years after surgery.

### 3.4 Complications

Table 2 illustrates all complications related to sEEG implantation and explantation, and any surgical intervention if done alongside with the explantation. Overall, no patients had any hemorrhaging with permanent effects from either sEEG surgery. Five patients had their sEEG electrodes adjusted during surgery, with none in the insulo‐opercular region or in the oblique trajectory. Patient 1 had minor hemorrhaging from a bone fiducial during implantation that resolved. One electrode in patient 5 had to be removed because of an anchor bolt plunge during implantation. Another electrode broke off in patient 18 and was retained beneath her galea. However, during explantation the electrode was able to be removed by drilling around the perforation and removing the area of bone in which it was embedded. This fractured electrode was a solitary electrode contralateral to the primary hypothesis in a 4‐year‐old patient with thin bone. Both electrodes were in the insulo‐opercular region.

**TABLE 2:** Summary of sEEG surgery complications

Patient ID	sEEG complications	Surgical complications	Permanent neurological deficits
1	None	Hemorrhage from bone fiducial during implantation (no permanent effects)	None
5	Anchor bolt of electrode V plunged into bone and had to be removed	None	None
18	A fracture of her right parietal sEEG electrode, Q21, during implantation, which was successfully removed during explantation	None	None

In terms of permanent neurological deficits, none of the patients developed any weakness, paresis, or other deficits that did not exist before the implantation and explantation. There were patients that had transient headaches that typically resolved after several days.

## 4 DISCUSSION

The insulo‐opercular region plays an important role in epileptic networks and there continues to be debate about the optimal methods for invasive monitoring in this region. In MR negative epilepsies in particular, localizing the EZ to the IO region can be considerably difficult.^15^ Scalp EEG recordings and semiology can provide some information but given the deep location of IO cortex are not always sufficient to detect IO region seizures without direct brain recordings.^24^ Thus, having safe methods for invasive monitoring of this region is crucial. We show here that sEEG exploration via an orthogonal or medial‐lateral approach is safe and often influences surgical planning.

Several other techniques have been described for invasive monitoring of the IO region. Open, image‐guided, depth electrode techniques are described, which can be employed as part of a craniotomy and grid electrode implantation.^25^, ^26^ Dissection and opening of the sylvian fissure is also described for orthogonal implantation of insular electrodes and implantation of grid electrodes directly on the opercular surface.^27^ The parasagittal transinsular apex electrode with perisylvian strips and grids has good spatial resolution and coverage of the insula. However, it is invasive as the depth electrode is placed parasagittally down the long axis of the insula through the insular apex and the sylvian fissure is dissected.^18^, ^19^ Additionally, using this approach the anteroinferior portion of the insula is not sufficiently covered.^19^

There are advantages and disadvantages of the orthogonal and oblique approaches to the IO region. The orthogonal approach enables simultaneous sampling of both the operculum and insula through a single short trajectory. One disadvantage of this approach is that the M2 branches of the MCA can make it difficult to access the inferior aspect of the first insular gyrus, which can necessitate use of an oblique trajectory. Another disadvantage of the orthogonal approach is that the position of insular recordings can depend on the orientation of MCA branches on preoperative vascular imaging. Although some surgeons always implant using a completely orthogonal approach to the insula with the assumption that electrodes will be deflected by arteries in their path, our practice is to place electrodes pseudo‐orthogonal (terminating outside the circular sulcus recording from the insulo‐opercular transition) when cerebrovasculature is not favorable for a direct orthogonal approach. The medial‐lateral oblique approach provides dense coverage of the insular gray matter but does not simultaneously record from the operculum. Given the length of insular oblique trajectories and the importance of distal electrode contacts, these trajectories are probably more prone to inaccuracies, although we did not see any instances of this in our series. Furthermore, it is important to note that with the standard orthogonal approach often only 1‐2 electrodes terminate in the insular gray matter, which is much fewer than the oblique approach.

Although our study focuses on the safety of insular‐opercular sEEG approaches, it is crucial to note that our findings also reveal that recordings from these electrodes are often relevant (see Figure 2). However, these electrodes should only be placed to test a well‐formulated hypothesis and not in the absence of a hypothesis. In other words, although the complication rates of these electrodes are low, they are likely not zero in large series. Each electrode trajectory should be carefully chosen and planned based on the necessity of insular coverage (i.e., whether the insula is a primary or secondary hypothesis) and the benefits for each individual patient.

A limitation of the existing literature is that most studies have been done in adult populations and although sEEG has also been shown to be effective in pediatric populations in Europe, very few studies have been done in the USA.^11^, ^12^ Unique features of pediatric patients, including decreased bone thickness, may alter the accuracy and safety of sEEG electrode implantations. For medial‐lateral insular oblique approaches which involve long trajectories where only the distal aspect of the electrode (which is most sensitive to entry point error and thin bone) records from the insular gray matter. In our evaluation, the safety of using sEEG placed into the insula and operculum in pediatric populations using various trajectories, including orthogonal and oblique, and found extremely low rates of complications.

Several limitations of the present study should be suggested. While these data show the safety of orthogonal and medial‐lateral oblique sEEG approaches to the IO region, the optimal approach between these two techniques is not resolved by our data. Further, lack of a direct comparison between sEEG approaches to the IO region and other approaches leaves optimal invasive monitoring modality an open question. With those limitations in mind, our results do show that sEEG approaches to the IO region can be safe and that open approaches are not necessary for invasive monitoring of this region.

It is also important to note that sEEG complication rates probably differ from center to center based on variation in technique. Some centers have reported up to 19% sEEG hemorrhage rates^28^ and other reports have suggested that sEEG hemorrhages may be more likely to result in a neurologic deficit than subdural‐related hemorrhages. It remains unclear if variations in technique results in complication rate variance, although it is logical to assume it does. For example, registration methods (face trace vs bone fiducials) may influence implantation accuracy and hemorrhage rate. Differences in sEEG imaging protocols may also play an important role.

## 5 CONCLUSIONS

Insulo‐opercular sEEG via orthogonal and medial‐lateral oblique is safe in pediatric epilepsy surgery patients. Our results also show that IO sEEG frequently influences pediatric epilepsy surgery resection plans. Future work may help to understand the relative risk‐benefit profile of different IO invasive monitoring approaches.

## AUTHOR CONTRIBUTIONS

All authors contributed to the collection and analysis of data.

## CONFLICT OF INTEREST

Dr. Abel is a consultant for and receives research funding from the Monteris Corporation.

## ETHICAL APPROVAL

An IRB‐approved protocol was used and given the retrospective and minimal risk nature of the study, consent requirements were waived.

## Data Availability

All data generated or analyzed during this study are included in this article. Further enquiries can be directed to the corresponding author.
